# Diagnostic potential of self-breast examination as an adjunct to mammography in females presenting with a breast lump: A review

**DOI:** 10.6026/973206300214908

**Published:** 2025-12-15

**Authors:** Shubham Rajesh Patil, Prathaben Maheshkumar Patel, Kosha Shah, Shreyanshi Satyambhai Vijakar, Rahi Sutaria, Nishant Kakkad

**Affiliations:** 1 Department of Anatomy, GMERS Medical College and Hospital, Sola, Ahmedabad-380060, Gujarat, India; 2 Department of Health, PHC Bamosana, Mehasana, Gujarat, India; 3 Department of Pathology, Narendra Modi Medical College, Ahmedabad, Gujarat, India; 4 Department of Pathology, GMERS Medical College, Himmatnagar, Gujarat, India; 5 Department of Medicine, GMERS Medical College and Hospital, Sola, Ahmedabad-380060, Gujarat, India

**Keywords:** Oncology, women's health, breast cancer, self-breast examination, mammography, early detection, resource-limited settings, health awareness, health education

## Abstract

Breast cancer is a leading cause of morbidity and mortality among women worldwide, with early detection playing a critical role in improving survival and quality of life. Mammography remains the cornerstone of breast cancer screening; however, its limited accessibility in many regions necessitates supportive diagnostic approaches. Self-breast examination (SBE) offers a simple, cost-effective, and empowering means for women to recognize early breast changes. When used as an adjunct to mammography, SBE can facilitate earlier presentation, particularly in younger women and resource-limited settings. Promoting awareness and proper education in SBE has the potential to strengthen breast cancer detection strategies and reduce diagnostic delays.

## Background:

Breast cancer constitutes a severe global health burden, with an estimated 2.1 million new cases diagnosed annually, accounting for nearly a quarter of all cancer cases in women [1]. The high prevalence of breast diseases, ranging from benign conditions to malignancies, is evidenced by the fact that approximately one in six women worldwide will undergo a breast biopsy in their lifetime [1, 2]. Risk factors such as advancing age, family history, reproductive patterns, hormonal therapies, and lifestyle choices amplify the urgency for effective and accessible early detection strategies [2]. Conventional screening modalities include mammography, clinical breast examination (CBE) and breast self-examination (BSE). While CBE and mammography are cornerstone techniques, their widespread implementation is often hampered by cost, infrastructure, and accessibility issues, especially in low-resource environments [3, 4]. In such contexts, BSE emerges as a critical, patient-empowering first line of defence. Therefore, it is of interest to elucidate the potential of SBE as a primary screening adjunct, aiming to inform public health initiatives and policies designed to improve early detection rates and outcomes for women globally.

## Awareness and prevention:

The escalating incidence of breast cancer globally highlights the profound importance of early detection. The level of awareness and societal attitudes towards breast cancer significantly influence participation in screening programs and, ultimately, survival outcomes [2]. Studies reveal a concerning disparity in the recognition of symptoms and risk factors, pointing to a pressing need for comprehensive public health education campaigns [5, 6]. Such initiatives are fundamental to primary prevention efforts and are particularly vital for younger women, who may perceive themselves as being at lower risk [5, 6]. Research by Memon *et al.* [6] identifies a troubling reluctance among young women to seek medical consultation upon discovering a breast lump, despite clinical recommendations. This hesitation, driven by factors like fear, embarrassment, and a lack of awareness, contributes significantly to delayed diagnoses and higher mortality rates, especially in Asian populations. Targeted interventions are urgently required to dismantle these barriers and promote proactive health-seeking behaviours in this demographic [6].

## Symptoms and risk factors:

Breast cancer can present with a variety of symptoms, including a palpable mass, nipple discharge (particularly bloody), changes in nipple appearance or retraction, skin redness, dimpling (peau d'orange), axillary lumps, and arm swelling [6, 7]. Unfortunately, deficient knowledge of these warning signs often leads to critical delays in seeking care [6, 7]. Common risk factors consistently identified across literature include a personal or family history of breast cancer, obesity, nulliparity, and alcohol consumption. Additional risk factors involve the use of oral contraceptives, hormone replacement therapy (HRT), and post-menopausal obesity (Figure 1 & 2 - see PDF).

## Investigations:

The diagnostic workup for a breast lump involves several modalities (Figure 3 - see PDF): Mammography, Ultrasound, Magnetic Resonance Imaging (MRI), Clinical Breast Examination (CBE), and Breast Self-Examination (BSE). In resource-constrained settings, the limited availability of mammography makes it an impractical tool for widespread primary screening. Consequently, BSE and CBE become not only more feasible but also essential components of early detection programs, underscoring the need for context-specific diagnostic strategies [8].

## Mammography:

It is the cornerstone of breast cancer screening, credited with reducing mortality by 20-25% through early detection [9, 10]. It is highly effective in identifying non-palpable masses and non-invasive cancers like ductal carcinoma in situ (DCIS). Major health bodies, including the American Cancer Society, recommend annual screening for women over 40, though guidelines have evolved to often recommend starting at 45 or 50 [11, 13]. Despite its efficacy, debates persist regarding its use, centered on concerns about over diagnosis and variable rates of advanced cancer detection in different populations [13, 14- 15].

## Breast Self-Examination (BSE):

BSE is a cost-effective, on-invasive technique that is particularly crucial in regions with limited healthcare access, such as rural India [16]. It empowers individuals to monitor their own breast health. Educational initiatives through training sessions, media campaigns, and guidance from healthcare providers are key to enhancing BSE awareness and proficiency [17]. The Canadian National Breast Screening Study (NBSS) provided robust evidence on the impact of proficient SBE, demonstrating a significant correlation between skilled self-examination practices and a reduced risk of breast cancer mortality [17, 18]. The technique involves both visual inspection and systematic palpation using the pads of the fingers in a circular motion, covering the entire breast and axillary area. While numerous guides exist, it is imperative for healthcare professionals to educate patients on the proper technique and to manage expectations regarding its reliability as a screening tool [19].

## Ultrasound and MRI:

Breast ultrasound is a safe, non-ionizing radiation modality excellent for differentiating cystic from solid lesions and guiding biopsies. However, it has lower sensitivity and specificity compared to mammography for overall cancer detection and is typically used as an adjunctive tool [20]. MRI offers superior sensitivity, especially in high-risk individuals and those with dense breast tissue, but its high cost limits its use as a primary screening method [20].

## Clinical Breast Examination (CBE): 

The American Cancer Society recommends CBE every three years for women in their 20s and 30s and annually for those 40 and older as part of a comprehensive health assessment [21].

## Management:

Increasing awareness and proficiency in BSE directly contributes to earlier detection, which expands treatment options (e.g., lumpectomy vs. mastectomy) and significantly reduces associated mortality and morbidity [22]. Furthermore, raising awareness about post-mastectomy reconstruction options can positively influence a patient's willingness to undergo treatment, improving overall psychosocial outcomes [22, 23].

## Additional Information:

Disclosures

## AI assistance disclosure:

The authors utilized an AI-assisted tool (OpenAI's GPT-4) during the manuscript preparation process to improve readability and language fluency. The authors are entirely responsible for the content and interpretation of the reviewed material.

## Limitations:

This review has several limitations. The 10-year publication window may not capture longer-term trends. The exclusive focus on female populations and English-language articles limits the generalizability of findings and may omit relevant research in other languages. Finally, restricting the search to four databases may have resulted in the omission of pertinent studies available elsewhere.

## Conclusion: 

The indispensable role of early detection in the fight against breast cancer is clear. While mammography remains the gold standard; its accessibility is not universal. In resource-limited settings, Breast Self-Examination emerges as a practical, empowering, and life-saving adjunctive tool. Data shows an urgent need for targeted educational initiatives to improve knowledge of risk factors, symptoms, and self-examination techniques, particularly among younger women. By addressing cultural and psychological barriers such as fear and embarrassment, we can foster a proactive approach to breast health, ultimately reducing global breast cancer incidence and mortality and improving the quality of life for women worldwide.
